# Constrained randomization and multivariate effect projections improve information extraction and biomarker pattern discovery in metabolomics studies involving dependent samples

**DOI:** 10.1007/s11306-015-0818-3

**Published:** 2015-06-02

**Authors:** Pär Jonsson, Anna Wuolikainen, Elin Thysell, Elin Chorell, Pär Stattin, Pernilla Wikström, Henrik Antti

**Affiliations:** 1 Department of Chemistry, Umeå University, S-901 87 Umeå, Sweden; 2 Department of Medical Biosciences, Pathology, Umeå University, S-901 87 Umeå, Sweden; 3 Department of Public Health and Clinical Medicine, Medicine, Umeå University, S-901 87 Umeå, Sweden; 4 Department Surgery and Perioperative Sciences, Urology and Andrology, Umeå University Hospital, S-901 87 Umeå, Sweden

**Keywords:** Metabolomics, Chemometrics, Dependent samples, Analytical drift, Run order design, Effect projections

## Abstract

Analytical drift is a major source of bias in mass spectrometry based metabolomics confounding interpretation and biomarker detection. So far, standard protocols for sample and data analysis have not been able to fully resolve this. We present a combined approach for minimizing the influence of analytical drift on multivariate comparisons of matched or dependent samples in mass spectrometry based metabolomics studies. The approach is building on a randomization procedure for sample run order, constrained to independent randomizations between and within dependent sample pairs (e.g. pre/post intervention). This is followed by a novel multivariate statistical analysis strategy allowing paired or dependent analyses of individual effects named OPLS-effect projections (OPLS-EP). We show, using simulated data that OPLS-EP gives improved interpretation over existing methods and that constrained randomization of sample run order in combination with an appropriate dependent statistical test increase the accuracy and sensitivity and decrease the false omission rate in biomarker detection. We verify these findings and prove the strength of the suggested approach in a clinical data set consisting of LC/MS data of blood plasma samples from patients before and after radical prostatectomy. Here OPLS-EP compared to traditional (independent) OPLS-discriminant analysis (OPLS-DA) on constrained randomized data gives a less complex model (3 versus 5 components) as well a higher predictive ability (Q2 = 0.80 versus Q2 = 0.55). We explain this by showing that paired statistical analysis detects 37 unique significant metabolites that were masked for the independent test due to bias, including analytical drift and inter-individual variation.

## Introduction

 A challenge in metabolomics is analysis of large sample cohorts or samples measured at different points in time since the analytical drift often introduces bias that obscures the analysis of data. This includes common situations such as, comparison of matched sample pairs (e.g. control versus case or pre versus post intervention) and matched sample series (e.g. individual subjects over time or the duration of a process). By default this is accounted for by randomization of the sample run order, with the assumption that systematic bias should be made independent of the biological variation of interest. NMR has thanks to its robustness so far been the leading tool in metabolomics for so called metabolome wide association studies (MWAS) (Chadeau-Hyam et al. 2010) as well as for combining data acquired over long periods of time or at multiple centers (Lindon et al. 2005). However, with the superior sensitivity of mass spectrometry (MS) there is a major incentive to solve this problem also for these techniques (Martin et al. 2014). Progress has been made within the field using mainly quality control (QC) sample strategies to make post analysis correction of the data to remove variation related to analytical drift (Dunn et al. 2011). This has proven efficient, but only solves part of the problem since some types of variation, caused e.g. by drops in sensitivity, or the fact that different compounds or compound classes with varying chemical properties are showing different drift patterns, are more or less impossible to correct for afterwards (van der Kloet et al. 2009). To account for or minimize such variation the actions must be taken prior to or during the analytical run. Actions prior to the analytical run is about creating a systematic scheme for the sample run order combining experimental design with randomization. Such approaches are simpler, more straightforward and mathematically more logical compared to actions during the actual run, and can if carried out correctly have a large positive impact on minimizing systematic bias in metabolomics data, or other analytical data. An example of an already existing and accepted approach is to use experimental design for creating sample run order schemes, e.g. to obtain a balanced distribution between controls and cases in separate batches or well plates. In the same way it would make sense to use a priori information about samples when creating analytical run order schemes when dealing with matched or dependent samples. The driving force for this is a higher quality output data more suitable for the following statistical analysis and evaluation. Thus in order to minimize the bias from the systematic instrumental drift on the comparison between matched samples, e.g. controls or cases or the same subject before and after an intervention, the most logical approach would be to keep them together as a separate item in the analysis. In this way the risk for confounding the biological variation of interest between the matched samples with the instrumental drift is minimized, something that is not guaranteed by full randomization. However, for this to have impact on the end results it requires that the subsequent statistical analysis also considers the sample matching or dependency. This can be compared to classical statistical approaches, where tests for paired and dependent samples exist in many shapes and forms. Nevertheless, in metabolomics studies the common analysis pipeline includes some type of pattern recognition based on multivariate statistics for elucidating metabolite signatures of biological relevance and predictive power. Most of these approaches, including all commercially available software, do not take into account paired or dependent samples in the analyses. Apart for being incorrect statistically it also diminishes the effect of a designed run order considering matched samples as attached items, as discussed above. There are however a few examples of multivariate approaches where samples dependency have been considered. (Keun et al. 2004; Lundstedt et al. 2010; Stenlund et al. 2009) Furthermore supervised multivariate approaches for the separation of between and within individual variation considering dependent subjects have been proposed (van Velzen et al. 2008; Westerhuis et al. 2010; Xu et al. 2014). Such approaches allow multivariate statistical analysis of paired or dependent samples and would thus benefit from a designed run order to minimize the confounding of instrumental drift bias with the investigated biological effect.

We present an approach for minimizing the influence of analytical drift on multivariate comparisons of matched samples in mass spectrometry based metabolomics studies. This is based on a “constrained” between and within matched item wise randomization procedure for sample run order, and a multivariate statistical analysis strategy allowing paired or dependent analysis based on orthogonal partial least squares (OPLS) (Trygg and Wold 2002) named OPLS-effect projections (OPLS-EP). The run order is created by considering matched samples as an attached item and randomization is carried out item wise, followed by a within item randomization to make sure that the within item order is random over the whole run. Multivariate statistical analysis on the acquired data is then carried out by means of OPLS-EP providing a dependent multivariate statistical analysis taking advantage of the OPLS method in terms of model interpretation. In summary we show how the combination of the suggested constrained randomization strategy and EP by OPLS largely facilitates interpretation and biomarker pattern discovery in metabolomics studies.

## Materials and methods

### Constrained randomization of runorder

**Fig. 1:**
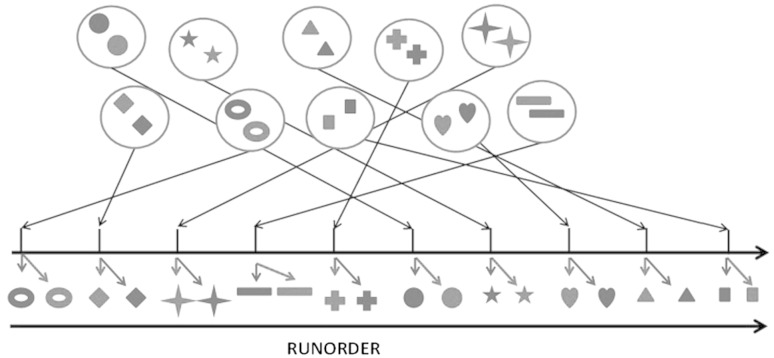
Scheme for constrained randomization of sample run order. The *same symbol* corresponds to matched/dependent samples. *Red symbols* pre intervention; *Blue symbols* post intervention. The procedure produces a runorder (*x*-axis) where matched or dependent samples are kept together as an item and randomization is done both between and within items (Color figure online)

The constrained randomization is based on a two-step procedure including a between and within item randomization. Thus, each sample is given two random numbers; where the first (RAND_IND_) is unique for each individual sample and the second (RAND_MATCH_) is shared for the individual samples in the same matched group or dependent item. The samples are then sorted in two steps; first upon RAND_MATCH_ and then upon RAND_IND_. This produces a run order where each item (matched group) is kept together in the analysis whereas the within item run order is randomized over the whole run, meaning that the only information that has to be un-blinded is which samples that are belonging to the same match group or dependent item (Fig. 1).

### Effect projections by means of OPLS (OPLS-EP)

In studies were the same (or matched) subject(s) has been characterized by the same variables before and after an intervention, the data can be seen as two matrices **X1**, including samples pre intervention, and **X2**, including samples from the same (or matched) subjects post intervention. PLS- or OPLS-discriminant analyses are common multivariate statistical tool within metabolomics to model and interpret the differences between the two sample sets **X1** and **X2**. However, these methods focus on finding the average difference between **X1** and **X2** and do not take into account the matched or paired sample information. This is similar to using an un-paired statistical test for defining significance in a data set consisting of paired samples. In contrast, our suggested method of EP by means of OPLS (OPLS-EP) models the variation in the “effect matrix” **XE**, formed by subtracting **X1** from **X2**, that differs from zero. **XE** then contains the effect of the intervention for each individual subject. In OPLS-EP the effect matrix **XE** is used together with a target vector of identity **y**, consisting of ones only. OPLS is then used to find the relation between **XE** and **y**. **XE** and **y** should not be mean centered prior to modelling but **XE** can be scaled as appropriate. Variables that have the largest absolute average in **XE** will have the largest impact on the model. If each variable in **XE** is scaled by dividing with its standard deviation the weight vector **w** will be proportional to the *t* values (from which *p* value are calculated) in a dependent (e.g. paired) *t* test for each variable.

### Data sets

#### Data set from Westerhuis et al. (2010)

The data in Table 1 was designed with the objective to model the differences between before and after treatment. In the data set “variable 1” is set to have a different response for males and females (+1 for men and +3 for women), “variable 2” has a common response (+2) for all subjects and “variable 3” shows no response with treatment. Furthermore, all variables were designed to contain large individual differences. Westerhuis et al. used the data to exemplify the features of their proposed ML-PLS-DA method for considering dependent samples by means of multivariate projections. Here we are using the same data set to compare and highlight the differences between the ML-PLS-DA, PLS-EP (an intermediate between ML-PLS-DA and OPLS-EP) and the OPLS-EP approach suggested by us. In this way we aim to stepwise clarify the benefits obtained by the EP and OPLS parts respectively.

**Table 1:** Simulated data adopted from Westerhuis et al. describing the same samples characterized by three variables before and after treatment

Sample	Subject	Gender	Variable 1	Variable 2	Variable 3
Before treatment					
1	1	Male	20	10	20
2	2	Female	18	12	17
3	3	Male	16	15	14
4	4	Female	14	16	11
5	5	Male	10	2	8
6	6	Female	9	3	5
7	7	Male	7	7	2
8	8	Female	7	7	8
9	9	Male	3	9	14
10	10	Female	2	9	17
After treatment					
11	1	Male	21	12	20
12	2	Female	21	14	17
13	3	Male	17	17	14
14	4	Female	17	18	11
15	5	Male	11	4	8
16	6	Female	12	5	5
17	7	Male	8	9	2
18	8	Female	10	9	8
19	9	Male	4	11	14
20	10	Female	5	11	17

#### Simulated instrumental drift

A simulated data set was constructed to study how different run order, magnitude of drift and type of statistical test affected the outcome of a metabolomics study. The data set consisted of 128 samples (64 matched objects; before and after intervention) characterized by one variable. In the “before” sample class the simulated variable were set to be normally distributed around a mean value of 100 while the mean value for the simulated variable in the “after” sample class was set to a value of 105. The standard deviation in both classes was set to the value 10. In addition an individual variation was added which was normally distributed around the value 0 with a standard deviation of 6. The full simulation was done using the following code in Matlab;


Xbefore = normrnd(100,10,64,1);



Xafter = normrnd(105,10,64,1);



IND_VAR = normrnd(0,6,64,1);



Xbefore = Xbefore+IND_VAR;



Xafter = Xafter+IND_VAR;


This produces a power of approximately 0.80 for a dependent (paired) Student’s *t* test (α = 0.05, 2-tailed) and approximately 0.70 for an independent (un-paired) Student’s *t* test (α = 0.05, 2-tailed). Furthermore a simulated instrumental drift (reflecting changes in sensitivity) was added to the data. Four different drift scenarios (a–d) were simulated;

- “*Slope*”, a slope ranging from 100 % to (100-drift) %.
- “*Step*”, a discrete step going from 100 % to (100-drift) % occurring after 64 samples.
- “*Wave*”, a sinus wave (one period) ranging from (100-drift) % to 100 %
- “Random”, as “Slope*”* but in random order.

Drifts ranging from 0 to 50 % were tested (unit steps). Prior to applying the simulated instrumental drift to the data the run order was set, using two different options; (1) traditional, or full, randomization (TR) or (2) constrained randomization (CR). The accuracy for both the dependent and independent *t* test was estimated for each type of drift (“Slope”, “Step”, “Wave” and “Random”), at each magnitude of drift (0–50 %) and for each type of randomization (TR or CR). The estimation of accuracy was done by creating the variable 10,000 times and test if there was a significant difference (α = 0.05, 2-tailed) between before and after sample(s) before and after applying the drift. The number of times that the tests displayed the same result before and after addition of drift was divided by the total number of tests (10,000) to get the estimated accuracy. The cause of error was studied by calculating; sensitivity, specificity, false discovery rate (FDR) and false omission rate (FOR) were the result before addition of drift was considered as the true condition and the result after addition of drift were seen as the test outcome. Accuracy was calculated as (Σ(True Positive) + Σ(True Negative))/Total number of tests, sensitivity as Σ(True Positive)/(Σ(True Positive) + Σ(False Negative)), specificity as Σ(True Negative)/(Σ(True Negative) + Σ(False Positive)), false discovery rate (FDR) as Σ(False Positive)/(Σ(True Positive) + Σ(False Positive)) and false omission rate (FOR) as Σ(False Negative)/(Σ(False Negative) + Σ(True Negative)).

### LC/MS radical prostatectomy data

#### Patients

Samples (n = 64) were selected from a clinical series of men treated with radical prostatectomy between February 2005 and September 2006 at the Department of Urology, Umeå University Hospital. Blood samples were drawn immediately before surgery and approximately 3 months after surgery. EDTA plasma was frozen and stored in −80 °C awaiting further analyses. All participants gave written consent for use of their blood samples in future research projects and the study was approved by the Research Ethics Board at Umeå University Hospital, Dnr 03-482.

#### Preparation of samples for LC/MS analysis

Plasma samples were allowed to thaw at room temperature just before extraction. To 100 µL of plasma, 900 µL of extraction solution (methanol/water (8:1)) with 4 internal standards (Val-Tyr-Val, Leu-Enk, Sulfadimetoxin, Reserpine) was added and the samples were vigorously extracted at a frequency of 30 Hz for 2 min using a MM301 vibration Mill (Retsch GmbH & Co. KG, Haan, Germany). After 120 min on ice, the samples were centrifuged at 14000 rpm for 10 min at 4 °C. A 250 µL aliquot of supernatant was transferred to a LC vial and evaporated to dryness.

#### Metabolite profiling

Untargeted metabolite profiling of plasma samples were performed by UHPLC-QTOFMSMS (Agilent 6540) equipped with a Kinetics 2.1 × 100 1.7u C18 column in positive mode 70–1700 m/z. The injection volume was 1 µL and column oven temperature was set to 40 °C. Constrained randomization was used to create the run order scheme so that the samples in the matched pairs were run adjacent to each other. Samples were analyzed by a 19 min revered-phase chromatography with gradient elution at 0.5 min/min flow rate from 99 % mobile phase H2O (0.1 % formic acid) to 99 % mobile acetonitrile (0.1 % formic acid).

#### Data processing and analysis

Data processing was done in MassHunter Qualitative Analysis software version B.06.00 (Agilent Technologies). Molecular Feature Extraction was performed through the “Find by Molecular Feature” function for a nontargeted approach. The processing generated a list with 797 putative metabolite peaks (m/z, retention time and peak area). Only peaks found in both subjects for a least 50 % of the pairs were used in the analysis. The processed data set thus consisted of 128 samples (64 pairs) characterized by 390 variables (metabolite peaks). The data set was analyzed using both an independent and a dependent Students’s *t* test of all individual variables. This was followed by multivariate analysis using the common OPLS-DA approach as well as the suggested OPLS-EP approach. The latter to obtain a multivariate model considering the sample dependency and to generate model scores revealing the magnitude of the metabolite profile change associated with the surgical intervention for each individual patient.

## Results

### Comparing ML-PLS-DA with OPLS-EP

**Fig. 2:**
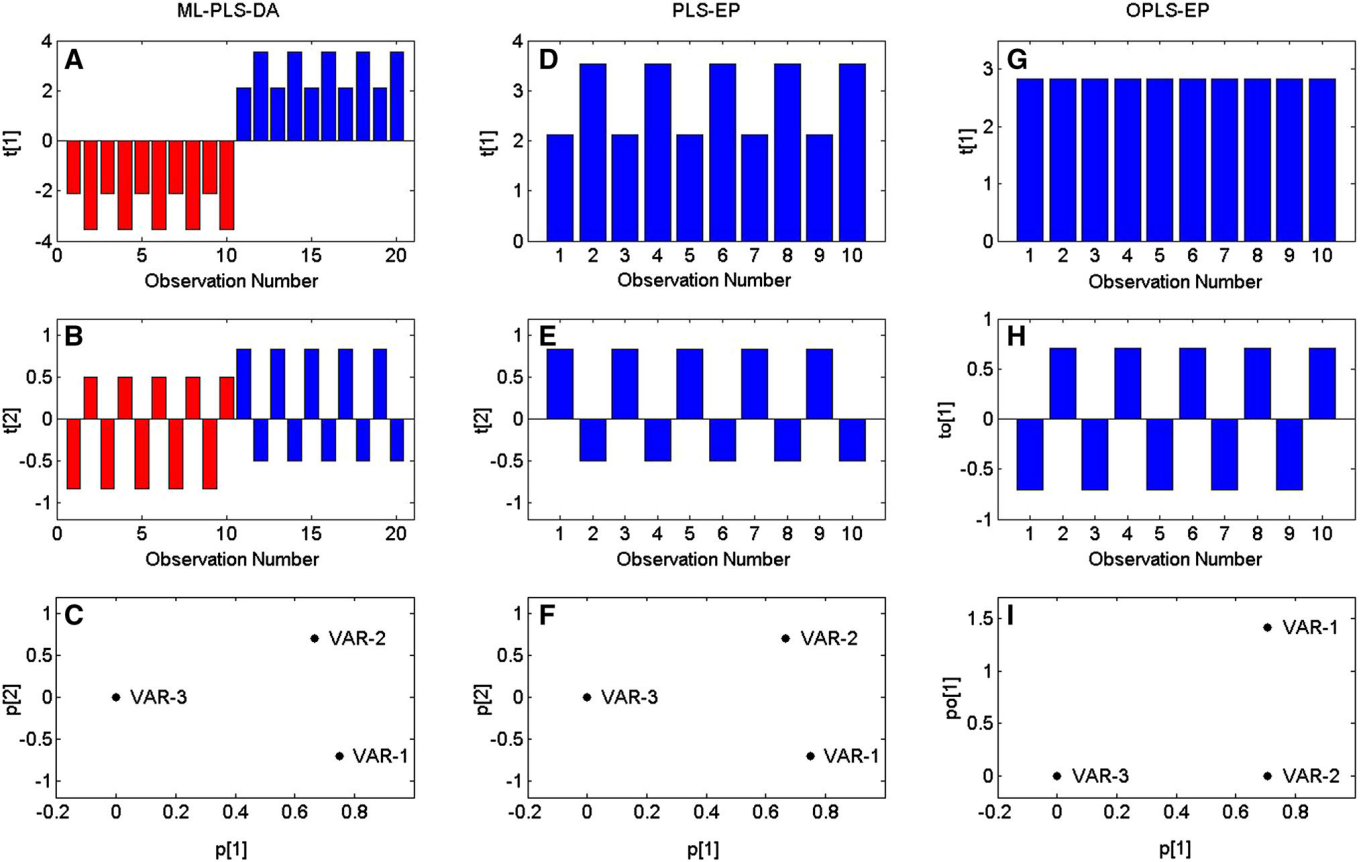
Comparison of model results from ML-PLS-DA (*left column* **a**–**c**), PLS-EP (*middle column* **d**–**f**) and OPLS-EP (*right column* **g**–**i**). All three models fits the data perfectly (R2 = 1), however the interpretation differs between them. Scores for ML-PLS-DA (**a**, **b**) showing a separation between pre minus post samples (*red bars*) and post minus pre samples (*blue bars*) mixed with gender differences (odd numbers: males, even numbers: females) in t[1] (**a**) and a compensatory effect in t[2] (**b**). The score plots (**d**, **e**) for PLS-EP are identical to the score plots from ML-PLS-DA except for that they do not contain a negative copy of each observation. Scores for OPLS-EP showing the effect of treatment in t[1] (predictive component, **g**) and the gender difference in to[1] (orthogonal component, **h**). Loadings for ML-PLS-DA (**c**), PLS-EP (**f**) and OPLS-EP (**i**) showing the mixed variable contribution in the two components for ML-PLS-DA and PLS-EP and the clear division of the different variable contributions into predictive and orthogonal components for OPLS-EP (Color figure online)

A comparison between OPLS-EP and ML-PLS-DA was done using the data presented by Westherhius et al. (Table 1). In order to make the comparison and development easier to follow an intermediate PLS-EP step was also applied to the same data to clarify the individual impact of the EP and the OPLS modeling respectively. For the effect projection approach an effect matrix **XE** is created by subtracting the pre-treatment data (pre) from the post-treatment data (post). As the response a constant vector **y** consisting of only ones are used and PLS or OPLS is used to find the relationship between **XE** and **y**. In ML-PLS-DA instead two classes “pre minus post” and “post minus pre” are constructed and PLS-DA is used to find the separatation between those classes. This means that in ML-PLS-DA the obtained X-matrix is [−**XE**;**XE**] and the response vector used is [−**y**;**y**]. For the presented data all three modeling approaches find a perfect fit to the response using two components (in this comparison data is not scaled). The only difference between ML-PLS-DA and PLS-EP is that in ML-PLS-DA there is an exact negative copy of each observation meaning that the number of observations is twice as many. The scores **T** from ML-PLS-DA corresponding to **XE** are identical to the scores **T** from PLS-EP, while the scores corresponding to −**XE** are negative copies of **T** from PLS-EP. Hence ML-PLS-DA contains redundant information in the observation direction. Variable weights **W** (not shown) and loadings **P** from ML-PLS-DA and PLS-EP are also identical providing the same interpretation. The difference between OPLS-EP and PLS-EP is the choice of multivariate method. OPLS separates the predictive and orthogonal variation into different components. In this example the orthogonal component **to**[1] is related to the gender difference while the predictive component **t**[1] shows the effect of treatment. PLS on the other hand mixes the two types of variation in both components, which is also the case for ML-PLS-DA. Model scores and loadings for ML-PLS-DA, PLS-EP and OPLS-EP are presented in Fig. 2. Scaling by the standard deviation will make the models of ML-PLS-DA different from models of PLS-EP if not a pooled standard deviation is used for ML-PLS-DA. If a pooled standard deviation is not used the most important variables (variables with large effect and small standard deviation) will be down scaled since the difference between the negative copies will contribute with large variance.

### Simulated instrumental drift

**Fig. 3:**
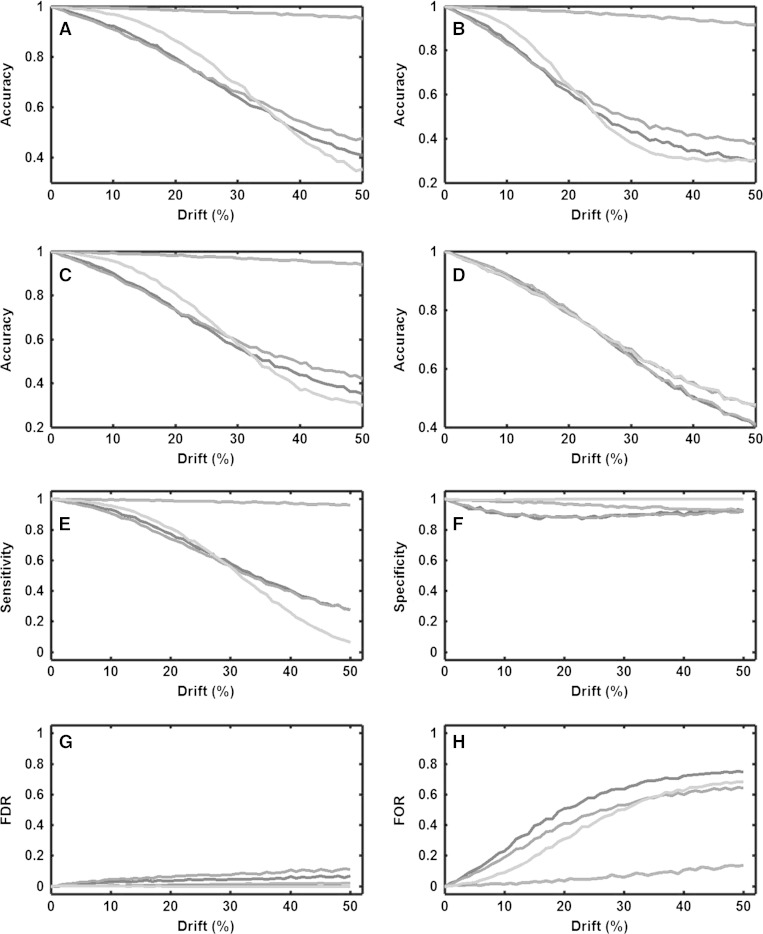
CR-DEP (*red line*), CR-IND (*turquoise line*), TR-DEP (*blue line*) and TR-IND (*green line*).In **a**–**d** four different types of drift were evaluated Slope (**a**), Step (**b**), Wave (**c**), and Random (**d**). In **a**–**c** the common trend is that CR-DEP is the only combination that maintains a high accuracy with increasing drift. Applying a random drift (**d**) none of the combinations can maintain a high accuracy with increased drift. In **e**–**h** four different measures of error are evaluated using the Slope drift. Sensitivity (**e**), Specificity (**f**), False discovery rate (FDR) (**g**) and False Omission Rate (FOR) (**h**). All four combinations show a fairly high Specificity (**f**) and fairly low FDR (**g**) independent of the magnitude of the drift. The major differences seen are that CR-DEP maintains a high Sensitivity (**e**) and a low FOR (**h**) with increasing drift as opposed to the other combinations. Although less evident, CR accounts for a lower FDR (**g**) and higher Specificity (**f**) as compared to TR (Color figure online)

In order to investigate how analytical drift, run order and choice of statistical method effect the accuracy, four different drift scenarios were tested, (i) a slope which simulates a continues drop in sensitivity during an analytical run, (ii) a step which simulates a clear discrete drop in sensitivity in the middle of an analytical run, (iii) a wave simulating a fluctuation (a sinus wave) of sensitivity during an analytical run and (iv) random noise. Two different run order approaches were tested (i) Traditional randomization (TR) and (ii) constrained randomization (CR). Furthermore, two variants of Student’s *t* test (dependent (DEP) and independent (IND)) were applied for evaluating the variable significance. Regardless of the pattern of the drift, the accuracy for both independent and dependent tests drops with increased magnitude of drift using traditional randomization (TR) (Fig. 3a–d). When using the constrained randomization (CR) a dependent *t* test can maintain the accuracy as long as the drift follows a pattern (Fig. 3a–c) but in case of random drift (Fig. 3d) the accuracy drops in the same way as when using the traditional randomization (TR) (Fig. 3a–c). When using constrained randomization (CR) together with an independent *t* test the accuracy drops more rapidly in comparison with traditional randomization (TR). The explanation to this is that the variation (caused by the drift) within the two groups are maximized by the constrained randomization (CR).

In Fig. 3e–h the cause of the drop in accuracy can be studied in detail by evaluating four different measures of error (using Slope drift). Here, it is clear that the major cause of drop in accuracy for CR-IND, TR-IND and TR-DEP is caused by increased number of false negatives since the false omission rate (FOR) increases dramatically and sensitivity decreases dramatically with increased drift, in comparison to changes in specificity and false discovery rate (FDR). CR-DEP was the only combination that maintained a high accuracy (Fig. 3e–h) and the reason for this is that it maintains a high sensitivity irrespective of the size of the drift (Fig. 3e). It is also clear that the problem associated with drift is that it gives rise to false negatives rather than false positives. Still, false positives are more prone to occur using TR in comparison to CR where the FDR is slightly lower.

### LC/MS radical prostatectomy data

**Fig. 4:**
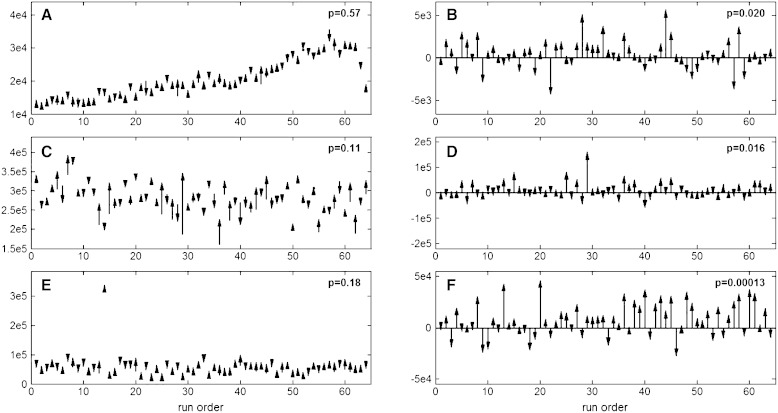
Examples of variables found significant by dependent but not by independent Student’s *t* test representing the three scenarios discussed in the text, (i) variables effected by analytical drift, (ii) variables showing a large variation between individuals and (iii) variables showing a deviating variation for one or a few patients (sample pairs). Peak area for the variable peak plotted against the run order for each matched pair (before and after surgery). The *length of the arrow* defines the magnitude of change in peak area between the matched samples, while the direction defines if there is an increase (*up*) or decrease (*down*) in peak area between the samples. *Left column* (**a**, **c**, **e**); uncorrected variables, corresponding to the independent test. *Right column* (**b**, **d**, **f**); the same variables after correction (peak area after − peak area before), corresponding to the dependent test. **a** Variable affected by analytical drift, p(independent test) = 0.75. **b** the same variable as in **a** after correction, p(dependent test) = 0.020. **c** Variable showing large variation between individuals, p(independent test) = 0.11. **d** The same variable as in **c** after correction, p(dependent test) = 0.016. **d** Variable showing large deviation for one individual, p(independent test) = 0.18. **e** The same variable as in **d** after correction, p(dependent test) = 0.00013

**Fig. 5:**
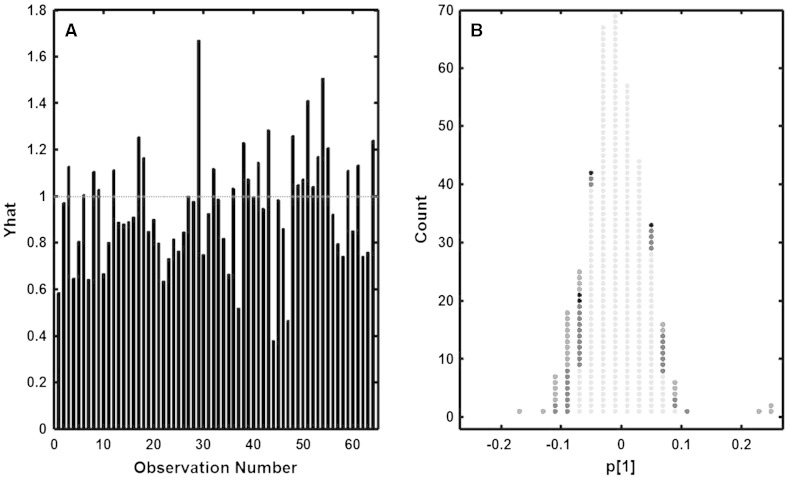
**a** Effect projection plot. Model estimation of the projected effect (Yhat). The dotted line (Yhat = 1) indicates the target value for the OPLS-EP model. Each individual patient corresponds to one observation number and the magnitude of the projected effect is given by the height of the corresponding black bar. Deviations from the value 1 for a specific patient indicate a larger (>1) or smaller (<1) metabolic effect (difference between after and before treatment) in the model direction (metabolic profile) associated with the surgical treatment. **b** Histogram of the predictive loading (p[1]) from the OPLS-EP model shows that variables significant in the both the independent and dependent univariate tests (*red dots*) and variables significant only in the dependent univariate test (*blue dots*) are among the most influential variables in the OPLS-EP model (high absolute loading value (|p[1]|)) for the effect of surgery. Interestingly variables found significant only by the independent univariate test (*black dots*) are found in connection to the other significant variables in the model loadings (although at the lower end of the ranking). Variables not found significant by any of the univariate tests (*grey dots*) are as expected ranked low by the OPLS-EP model (low absolute loading value (|p[1]|). However a few exceptions can be found indicating that the multivariate model is picking up variables as influential to the model that were not defined as significant by univariate statistics (Color figure online)

The processed LC/MS data from the radical prostatectomy study was subjected to multivariate data analysis by means of OPLS-DA and OPLS-EP. Prior to OPLS-DA modeling the X-variables (LC/MS) where mean centered and scaled to unit variance. For OPLS-EP only scaling to unit variance was performed. The predictive ability of the models (Q2) was estimated using a 64-fold cross validation procedure (Wold 1978) (leaving one matched pair out). The predictive ability Q2 is a statistical measure of the stability of the models. For OPLS-DA this corresponds to the stability of the between class difference and for OPLS-EP to the stability of the projected effect. Each variable in **X** used in the OPLS-DA model was scaled by the pooled standard deviation for each variable for the two classes (pre and post surgery) while the variables in **XE** used in OPLS-EP was scaled by the standard deviation of each variable. Comparing the models based on the two methods (OPLS-DA and OPLS-EP) it was seen that the OPLS-EP model was less complex in comparison to the OPLS-DA model regarding the number of significant components, 3 (1 predictive + 2 orthogonal) for OPLS-EP versus 5 (1 predictive + 4 orthogonal) for OPLS-DA. The reason for this being that the OPLS-DA model needs to handle more orthogonal variation (individual variation caused by a higher number of samples as well as variation caused by analytical drift since the sample dependency is not considered). Both methods showed a similar description of the response variation (pre versus post surgery) (R2Y; OPLS-DA: 0.92, OPLS-EP: 0.94) however the OPLS-EP model gave a higher predictive ability of the response variation (Q2; OPLS-DA: 0.55, OPLS-EP: 0.80). This suggests that there is a clear structure in the data associated with the sample dependency. To verify this all variables were tested for univariate significance using *t* tests (dependent and independent). For the independent *t* test 33 of the 390 variables (8.4 %) were found significant (*p* < 0.05) whereas for the dependent *t* test 66 (16.9 %) variables were found significant. 29 (7.4 %) variables were considered significant by both tests while only 4 (1.0 %) variables were found to be uniquely significant by the independent test and 37 (9.5 %) by the dependent *t* test. The 37 variables found uniquely significant by the dependent *t* test were further scrutinized and it was seen that they could be divided into three major categories being, (i) variables effected by analytical drift, (ii) variables showing a large variation between individuals and (iii) variables showing a deviating variation for one or a few patients (sample pairs) (Fig. 4). Interpretation or ranking of variables importance for OPLS and PLS models can be done in different ways, interpreation of weights (**w**), loadings (**p**), regression coefficents, selectivity ratio (Rajalahti et al. 2009) or VIP (variable influence on projection) (Galindo-Prieto et al. 2014). The different strategies will give slightly different ranking of variables (but that is outside the scope of this article). We here choose to use the predictive loading (**p[1]**) for the OPLS-EP model and compare that to the outcome of the the univariate test (Fig. 5b). From the figure it can be seen that the OPLS-EP predictive loading (**p[1]**) does not correlate perfectly with the significance ranking of the univariate test. However there is a clear trend that the significant variables from the univariate test are found among the most influential variables in the OPLS-EP model.

In addition, the OPLS-EP model provided a straightforward visualization of the individual responses to the surgical intervention (radical prostatectomy) as an effect projection (Yhat) focusing on the change in the intervention specific metabolic signature of the dependent samples (Fig. 5a). Here, the effects could be compared to the target value (Yhat = 1) in order to detect individuals or subgroups of individuals with a deviating metabolic response to treatment.

## Discussion

Our results show that the suggested constrained randomization procedure is advantageous in studies including dependent or matched samples. It is evident that by keeping dependent samples together as an item in the analysis instrumental drift bias will be practically un-confounded with the biological variation of interest, i.e. within item variation, which in turn facilitates interpretation and biomarker discovery by means of statistical analyses that consider the sample dependency. In a way this is challenging the common statistical assumption of a fully randomized run order for handling potential confounding bias. However, it is clear that a traditional randomization approach will not be able to completely reduce the bias introduced by instrumental drift over the analytical run, due to the fact that dependent samples can end up at completely different ends of the analytical run making it difficult, if not impossible, to separate the true biological effect from the instrumental drift variation. A criticism to be expected towards the suggested constrained randomization is that it complicates the design of fully blinded studies. However, the only difference between a fully blinded study and a study based on the suggested approach is that the sample matching or dependency has to be known prior to analysis. Apart from that the approach is completely based on full randomization both between and within dependent items so no information in relation to the biological question of interest, e.g. control/case or pre/post intervention, is necessary to reveal. We thus consider the benefits of the suggested constrained randomization approach over the traditional to be so significant that it is by all means justified to alter the traditional randomization strategy to better suit the study design. After all the aim of metabolomics or other bioanalytical studies is to generate data of high quality that allows addressing pre-defined questions and hypotheses with high reliability and to obtain this it makes sense to at least start off with the best possibilities in terms of design of the study and run order prior to the actual analysis step.

Common practice regarding data analysis in metabolomics studies is to use some type of multivariate statistical approach to build predictive models of, and extract biomarker patterns from, the whole metabolite profile generated in the sample analysis. For this the so called multivariate projection methods, e.g. PLS or OPLS (Trygg and Wold 2002; Wold et al. 2001), have become very popular since they offer a high level of transparency in the interpretation of the calculated models. Especially the versions of the methods focusing on discriminant analysis (DA) are widely used since many applications involve searching for differences between pre-defined sample classes where matched or dependent samples are frequently occurring. Although this is the case almost all applications of the DA methods in metabolomics, and related fields, are focusing on multivariate discrimination of sample classes by comparing the class averages and thus not taking the sample matching or dependency into account. So, in cases where it would have been a given to use a paired or dependent statistical test in univariate statistics this is far from obvious in the multivariate case. This issue has been addressed previously and feasible multivariate approaches to resolve it has been presented (van Velzen et al. 2008; Westerhuis et al. 2010; Xu et al. 2014). However, still the majority of the data analyses presented in metabolomics studies are based on comparing class averages instead of considering the existing sample matching or dependency. Our addition in terms of the presented effect projection approach coupled to OPLS is here shown to include a number of useful features for the analysis of omics data sets including matched or dependent samples. By using the constructed effect matrix **XE**, containing the individual effects in the dependent or matched samples, for multivariate projections by means of OPLS against a constant vector a paired or dependent multivariate test is obtained where the individual subject’s effects different from zero can be statistically tested in a comparable way to a traditional univariate hypothesis test. In addition, this multivariate hypothesis test comes with all the additional features offered by the OPLS method, such as visualization, interpretation and validation of the results as well as the possibility to make predictions of the effects for new samples based on the existing model. In addition, it is from a user point of view completely straight forward to perform this in any multivariate software offering the OPLS method, which importance should not be underestimated. The interpretation feature is one of the main benefits of the OPLS-EP method, since it allows focusing on the effect related variation in the predictive OPLS component, while the orthogonal variation is modelled and can be interpreted separately in additional orthogonal components. Compared to the ML-PLS-DA by Westerhuis et al., as well as an intermediate PLS-EP approach used in the comparison, the facilitated interpretation offered by OPLS-EP is clear from our results and should be seen as a great asset of the proposed method. In that respect ML-PLS-DA and PLS-EP suffers from mixing of predictive and orthogonal variation in the model components, which to some extent confuses the interpretation. In addition it was also seen that ML-PLS-DA and PLS-EP, due to the way the data is pre-treated (mean centered), provides model scores with a mirror pattern that creates artificial between sample differences. In this case it creates a difference between male and female subjects before treatment that is not present in the data (Fig. 3). These problems are resolved by the OPLS-EP approach by showing the magnitude of the individual effects in the predictive component while revealing other correct systematic differences of interest in the orthogonal components. In addition, the EP approach of applying a multivariate projection method on the effect matrix (**XE**) is a natural extension of a dependent univariate significance test, which makes it intuitively more graspable as compared to other working multivariate versions including ML-PLS-DA.

An issue, which is often discussed in clinical applications of omics studies, is how to be able to detect or define subpopulations of disease at diagnosis or how to be able to monitor the individual response to an intervention or treatment based on some biochemical signature or pattern (Trygg et al. 2007). We hypothesize that the proposed OPLS-EP method can be a tool to aid in this development of new and more informative molecular diagnostics. The basis for this hypothesis is that the multivariate effect projection score (Fig. 5a) will contain information that can be used to detect subgroups either by visual detection and statistical significance testing or by correlating to other sources of data, e.g. gene mutations, clinical outcome, survival, etc. (Malone et al. 2014). Regarding response to an intervention or treatment it would be obvious to use the individual EP to define which subjects that show a statistically significant effect and which do not. This response grouping could be used as a means by itself to distinguish responders from non-responders, or to define a response continuum, but could also be used to correlate against other data sources to get more insight into what causes these differences in response. In our presented example of patients before and after radical prostatectomy surgery it would for instance have been interesting to correlate the effect projection scores against the future clinical outcome in terms of disease relapse, in order to obtain a molecular signature post surgery that can be used to base further treatment decisions on. Furthermore, subgroups of other than clinical origin would also be valuable to detect and evaluate. This could then work as a tool to detect unknown biases, information that could be useful in the design of new studies e.g. for future matching of samples. It is also of importance to investigate the usefulness of the orthogonal variation in the OPLS-EP models. One interesting clinical application could be to study if it is possible to separate the effect of a specific treatment and possible adverse effects so that the treatment effect is modelled in the predictive model component while the adverse effect or effects are modelled in the orthogonal counterparts.

As shown by our results the constrained randomization approach is vital for the OPLS-EP method to perform optimally when analytical drift is present. The reason for this being that the constrained randomization as opposed to the traditional fully randomized counterpart makes sure to minimize the influence of analytical drift on the between dependent sample differences. These issues have earlier been addressed by performing post correction of the acquired data in order to correct for the bias introduced by the instrumental drift (Dunn et al. 2012; Kamleh et al. 2012; van der Kloet et al. 2009). Although sufficient and sophisticated, the need of major post correction methods is usually a sign of suboptimal procedures prior to and during the analytical run. In our mind post correction methods should be a tool to fine tune the quality of the data to allow higher sensitivity analyses for e.g. biomarker discovery. Thus we believe that such post correction could be valuable as a complement to the constrained randomization approach and maybe create means to further optimize the quality of the data. The aim of most metabolomics studies is to some extent linked to biomarker discovery, meaning identification of metabolites or patterns of metabolites that can be used for diagnostic or prognostic purposes, target identification or as a means to get a deeper understanding of the studied biological process. In this paper we show that when the study design is set up to contain dependent or matched samples it is of value for the sensitivity of the biomarker discovery to use the constrained randomization approach for minimizing drift bias combined with a data analysis approach taking the sample dependency or matching into account, e.g. paired Student’s *t* test or OPLS-EP. In the example including radical prostatectomy patients pre and post surgery it was seen when comparing the conventional OPLS-DA approach to the OPLS-EP approach that the latter provides a much more reliable and sensitive output in terms of a less complex model due to minimization of analytical drift bias as well as a more predictive model, which is directly associated with the detection of a higher number of significant metabolites, i.e. potential biomarkers. This was also verified and further investigated in detail by comparing the output of an unpaired versus a paired Student’s *t* test on the variable significance. We saw that the paired test was able to find 37 significant variables (metabolite peaks) that were masked for the independent test due to different sources of bias, here identified as instrumental drift, large inter-individual variation and few largely deviating individuals (Fig. 4). We also showed that these 37 significant metabolite peaks were ranked among the top influential variables in the multivariate OPLS-EP model, which validates the value of OPLS-EP from a biomarker detection perspective (Fig. 5b). The identified sources of bias have a common denominator in that they cause the baseline samples, here pre surgery, to have different starting points, making the independent analyses severely suboptimal since they focus on the difference between group averages. This is also obvious when using e.g. OPLS-DA (independent analysis) for analyzing dependent data, which is the common case in most studies today. In cases where the bias is of biological origin, i.e. baseline samples have different starting points due to biological differences the dependent OPLS-EP approach will resolve this problem on its own, independent of randomization method. However, when the bias is due to analytical drift the use of the constrained randomization prior to the OPLS-EP analysis will be absolutely crucial. In our mind this raises a question whether it would be a preferred strategy to work more according to the procedure *sample matching*—*constrained randomization*—*OPLS*-*EP* even in cases where there is no true sample dependency, at least in the biomarker discovery phase. This is already the case in many clinical studies where samples are matched according to e.g. gender, age, and time in storage. We believe that this type of matching could be extended further to include other types of studies but also be made more efficient and correct by including more data describing the samples. For instance in the case of clinical studies or in biobanks a wealth of parameters are usually collected describing the samples or subjects. By taking advantage of this information multivariate tools like principal components analysis, clustering techniques or similar could be utilized for a more sophisticated sample matching. Our hypothesis is that by using this matching as a means for constrained randomization followed by OPLS-EP we will obtain a sensitive tool for biomarker pattern discovery. The fact that the matched samples are not truly dependent might be to challenge existing statistical rules, however if the sensitivity for detecting biomarkers is higher and that proper validation of detected biomarkers in new studies and settings verifies the value, then this development serves a purpose making it worthwhile to consider as a preferred method of choice.

Futurewise, we foresee many interesting applications for the proposed procedure that we aim to explore in more detail. For instance we hypothesize that it can be of great value for solving problems related to drift in data from samples measured at different points in time, e.g. exploratory study and follow up study for validation/verification, samples measured at different labs, e.g. multi-center studies, as well as the high reward problem of combining and interpreting data from the same samples measured at different analytical platforms or originating from different sources within an individual or system. From a clinical perspective we believe that the suggested methodology paves way for higher sensitivity in detection of subgroups among individuals, e.g. in terms of disease aggressiveness or response to treatment. Furthermore we envisage that the methodology will support the development and application of clinical studies designed as interventions to further increase sensitivity in detection of biomarkers and disease subgroups.

## Concluding remarks

In conclusion the presented results show how the combination of the constrained randomization strategy and multivariate analysis by means of EP by OPLS can increase the sensitivity and simplify the interpretation of biomarker patterns in metabolomics studies of dependent samples. Constrained randomization and OPLS-EP should be seen as novel and useful additions to the field of metabolomics, as well as in general to other fields involving multivariate characterization and comparison of dependent samples.
